# Optimized Hounsfield Units Transformation for Explainable Temporal Stage-Specific Ischemic Stroke Classification in CT Imaging

**DOI:** 10.3390/jimaging11120423

**Published:** 2025-11-28

**Authors:** Radwan Qasrawi, Suliman Thwib, Ghada Issa, Ibrahem Qdaih, Razan Abu Ghoush, Hamza Arjah

**Affiliations:** 1 Department of Computer Science, Al Quds University, Jerusalem P.O. Box 20002, Palestine; sthwib@staff.alquds.edu (S.T.); gissa@staff.alquds.edu (G.I.); razan.ghoush@staff.alquds.edu (R.A.G.); 2 Department of Computer Engineering, Istinye University, Istanbul P.O. Box 34010, Turkey; 3 Department of Medical Imaging, Al Quds University, Jerusalem P.O. Box 20002, Palestine; 4 Department of Radiology, Istishari Arab Hospital, Ramallah P.O. Box 20002, Palestine; 5 Faculty of Medicine, Al Quds University, Ramallah P.O. Box 20002, Palestine

**Keywords:** ischemic stroke, computed tomography, hounsfield unit, image enhancement, neural networks, explainable AI

## Abstract

Background: The early and accurate classification of ischemic stroke stages on computed tomography (CT) remains challenging due to subtle attenuation differences and significant scanner variability. This study developed a neural network framework to dynamically optimize Hounsfield Unit (HU) transformations and CLAHE parameters for temporal stage-specific stroke classification. Methods: We analyzed 1480 CT cases from 68 patients across five stages (hyperacute, acute, subacute, chronic, and normal). The training data were augmented via horizontal flipping, ±7° rotation. A convolutional neural network (CNN) was used to optimize linear transformation and CLAHE parameters through a combined loss function incorporating the effective measure of enhancement (EME), peak signal-to-noise ratio (PSNR), and regularization. the enhanced images were classified using logistic regression (LR), support vector machines (SVMs), and random forests (RFs) with 25-fold cross-validation. Model interpretability was evaluated using Grad-CAM. Results: Neural network optimization significantly outperformed static parameters across validation metrics. Deep CLAHE achieved the following accuracies versus static CLAHE: hyperacute (0.9838 vs. 0.9754), acute (0.9904 vs. 0.9873), subacute (0.9948 vs. 0.9825), and chronic (near-perfect 0.9979 vs. 0.9808). Qualitative interpretability analysis confirmed that models focused on clinically relevant regions, with optimized enhancement producing more coherent attention patterns than static methods. Parameter analysis revealed stage-aware adaptation: conservative enhancement in early phases (slope: 1.249–1.257), maximized in subacute (slope: 1.290–1.292), and restrained in the chronic phase (slope: 1.240–1.258), reflecting underlying stroke pathophysiology. Conclusions: A neural network-optimized framework with interpretability validation provides stage-specific stroke classification that achieves superior performance over static methods. Its pathophysiology-aligned parameter adaptation offers a clinically viable and transparent solution for emergency stroke assessment.

## 1. Introduction

Ischemic stroke is a leading cause of death and long-term disability worldwide, accounting for the majority of all stroke cases. It occurs when a thrombus or embolus obstructs cerebral blood flow, leading to ischemia, neuronal injury, and, if untreated, irreversible infarction [1]. Patient prognosis depends heavily on the speed and accuracy of diagnosis, as the benefits of reperfusion therapies, such as intravenous thrombolysis and mechanical thrombectomy, diminish rapidly after symptom onset [2,3]. Accurate classification of the stroke stage is therefore essential not only for determining treatment eligibility but also for predicting patient outcomes and guiding secondary prevention strategies [4,5].

Computed tomography (CT) is the most widely used initial imaging modality for stroke assessment due to its speed, accessibility, and ability to exclude hemorrhage [6]. While non-contrast CT (NCCT) is the standard first-line imaging technique, its sensitivity for detecting early ischemic changes is limited, particularly within the first few hours. Conventional interpretation relies heavily on qualitative visual assessment, which can be subjective and vary based on reader experience [7,8,9]. To address this, quantitative imaging biomarkers derived from Hounsfield Unit (HU) measurements have been investigated to improve detection sensitivity and provide a more objective diagnostic basis [10]. Hounsfield Units measure tissue attenuation on CT and can reveal subtle changes in brain parenchyma related to ischemia, edema, and hemorrhagic transformation.

The temporal evolution of ischemic stroke follows a predictable pathophysiological progression, with corresponding changes in CT attenuation [11]. The hyperacute stage may show only slight hypoattenuation; the acute and subacute phases exhibit more pronounced changes; and chronic infarcts display well-defined areas of low attenuation and tissue loss [12]. By quantifying these attenuation changes, researchers have sought to develop stage-specific classification systems that overcome the limitations of visual assessment.

Several studies have explored the potential of HU-based metrics for stroke classification. Mokin et al. demonstrated that the ratio and difference in HU values between affected and contralateral brain regions correlated strongly with ASPECTS-based assessments (correlation coefficient: 0.71) [10]. Reidler et al. expanded on this by combining relative HU with ASPECTS scoring, reporting area under the curve (AUC) values of 0.80 in test sets and 0.74 in validation sets, with sensitivities up to 75% and specificities above 82% [13]. Said et al. proposed specific HU thresholds to differentiate normal brain tissue from hyperacute, acute, subacute, and chronic ischemic stages, reporting 90% classification accuracy [14].

Other research has focused on optimizing CT window width and level settings to enhance the detection of early ischemic changes. Lev et al. showed that adjusting these settings improved sensitivity from 57% with standard settings to 71% with optimized values, while maintaining 100% specificity [15]. Mainali et al. confirmed that using standardized “stroke windows” significantly increased early ischemia detection rates. However, these methods are relatively simple to implement but their performance can vary depending on scanner type and institutional protocols [16].

Beyond traditional thresholding and visual optimization techniques, advanced computational approaches have emerged. Cheng et al. utilized CT angiography source imaging combined with HU attenuation and automated ASPECTS scoring to predict hemorrhagic transformation, achieving AUC values above 0.70 [17]. Khalilzadeh et al. applied relative mean enhancement decay modeling to discriminate between hemorrhage and iodinated contrast, achieving a sensitivity of 93% and specificity of 91% [11]. Radiomics-based analyses, such as that of Yao et al., have demonstrated high discriminatory power for estimating time since stroke onset, with validation AUC values exceeding 0.97 [18]. More recently, deep learning approaches applied to large CT datasets, as in the work of Inamdar et al., have achieved multi-stage classification accuracies above 92%in multi-center data and nearly 99%in single-center settings [19].

Despite notable progress in using HU measurements to classify ischemic stroke stages, timely and accurate identification remains critical, as treatment decisions depend heavily on the stage of stroke progression. Non-contrast CT, the most common first-line imaging tool, is widely accessible but has limited sensitivity for detecting early and subtle ischemic changes. Furthermore, raw HU values often vary across scanners and patients, complicating the reliable distinction between different temporal stages of ischemia.

Most existing studies have relied on retrospective datasets or have not implemented standardized HU transformations, limiting their reproducibility and hindering translation into clinical practice. Consequently, many promising methods remain confined to research settings rather than supporting real-time patient care.

This study addresses these challenges by introducing an optimized HU transformation framework specifically designed to highlight subtle ischemic changes and improve stage-specific classification. By standardizing HU values, our approach aims to enhance diagnostic accuracy, support timely clinical decision-making, and facilitate the integration of advanced imaging analysis into routine stroke management. This work seeks to bridge the gap between technical innovation and clinical application, with the aim of improving patient outcomes and expanding access to reliable stroke diagnosis.

## 2. Methodology

### 2.1. Study Design and Objectives

This study was designed as a retrospective diagnostic accuracy investigation with aimed at improving the classification of ischemic stroke stages using CT imaging. We focused on refining Hounsfield Unit (HU) transformations to ensure consistent performance across different CT scanners and to adapt to the dynamic changes in brain tissue that occur throughout stroke progression. Traditional approaches are often limited because HU values for gray matter (GM) and white matter (WM) vary significantly between scanners and also change as a stroke evolves from the hyperacute phase to the acute, subacute, and chronic stages. Standard linear transformation methods frequently fail to maintain clear GM–WM differentiation under these varying conditions.

**Figure 1:**
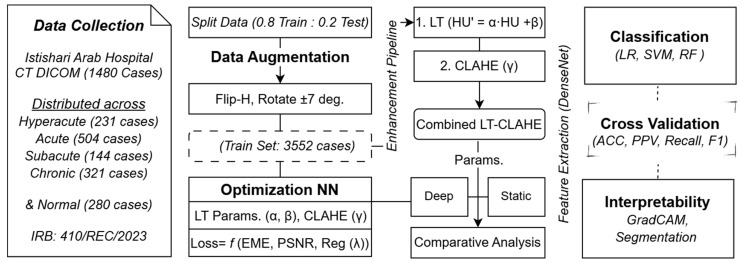
Complete Workflow for Stroke Classification Framework Using Neural Network-Optimized Image Enhancement Pipeline.

To address these limitations, we developed a method that dynamically optimizes HU transformations dynamically and integrates them with a non-linear image enhancement process to improve the visibility of stroke-related changes. The primary objectives were threefold: (1) to develop a neural network-based approach for dynamically determining optimal linear transformation parameters that maximize GM-WM separation in individual stroke images; (2) to identify optimal parameter ranges for each stroke temporal stage (hyperacute, acute, subacute, chronic) through neural network training; and (3) to integrate optimized linear transformation with Contrast Limited Adaptive Histogram Equalization (CLAHE), using neural networks to simultaneously optimize both transformation parameters and thereby enhance final image quality and stroke classification accuracy. The methodology structure is illustrated in Figure 1, which depicts the complete workflow from data collection through enhancement, classification, and final validation.

### 2.2. Study Population and Dataset

This study utilized CT scans from 68 patients from the Istishari Hospital, comprising a total of 1480 individual scans. Our cohort comprised 72% male and 28% female patients, with a mean age of 65 years (range: 26–83 years). The cohort included 12 patients in the hyperacute stage (231 cases), 22 in the acute stage (504 cases), 10 in the subacute stage (144 cases), and 14 in the chronic stage (321 cases), along with 10 normal controls (280 cases). All stroke diagnoses were confirmed through both clinical evaluation and neuroradiological interpretation.

Following the initial data collection, the training dataset was augmented to enhance model robustness and generalization. We applied horizontal flipping and rotation by ±7 degrees to the training data, expanding the training set from 1184 cases (80% of total) to 3552 cases. This augmented dataset was then processed through the neural network-based optimization pipeline, which dynamically determined the optimal enhancement parameters for each temporal stage.

The dataset captures distinct imaging characteristics across stroke progression stages. Hyperacute cases (within 6 h) represent the earliest phase where CT changes are extremely subtle and often closely resembling normal brain tissue. Acute cases (6–24 h) show emerging hypodensity as the infarcted area becomes apparent. Subacute cases (1–14 days) exhibit noticeable hypodensity and swelling that clearly demarcate the affected tissue. Chronic cases (>14 days) present well-established lesions characterized by gliosis and volume loss. Normal controls were selected from individuals who underwent CT for non-neurological reasons and showed no evidence of cerebrovascular disease. Scans were excluded if image quality was compromised by motion or artifacts, if hemorrhage was present, or if essential acquisition metadata were unavailable.

### 2.3. Image Acquisition and Preprocessing

To ensure heterogeneity in acquisition protocols and technical parameters, CT scans were acquired from three different scanners: two Philips Incisive 128-slice scanners manufactured by Koninklijke Philips N.V. (Amsterdam, The Netherlands), and one Toshiba (Canon) Aquilion 128 CT scanner from Canon Medical Systems Corporation (Ōtawara, Tochigi, Japan). All scanners operated at 120 kVp and 300 mAs with CTDI values ranging from 52.3 to 56 mGy. Brain imaging protocols were standardized across scanners with window level 40 HU, window width 80 HU, and 512 × 512 matrix size, though reconstruction algorithms varied (AIDR 3D Integrated for Toshiba, iDose for Philips). Slice thickness ranged from 0.5 mm to 5 mm depending on scanner capabilities, with helical pitch between 0.4 and 0.5. This variability was intentional, to test the robustness of our proposed approach and to reflect real-world clinical conditions. All imaging data were fully de-identified in accordance with institutional ethical guidelines and stored in standard DICOM format for subsequent analysis.

To minimize technical bias, several preprocessing steps were applied uniformly across all scans. Raw DICOM pixel data were first converted to Hounsfield Units using the scanner-specific rescaling parameters stored in the DICOM headers. Images were then resampled to a consistent matrix size of 512 × 512 pixels to ensure uniform spatial resolution. Standard brain windowing was applied (center = 35 HU, width = 70 HU) to optimize brain tissue contrast. This preprocessing pipeline preserved the original HU values necessary for subsequent linear transformation while providing a consistent input format for the optimization model. Normalization to an 8-bit range (0–255) was performed after linear transformation to prepare the images for CLAHE enhancement.

### 2.4. Stage-Specific Parameter Optimization

**Figure 2:**
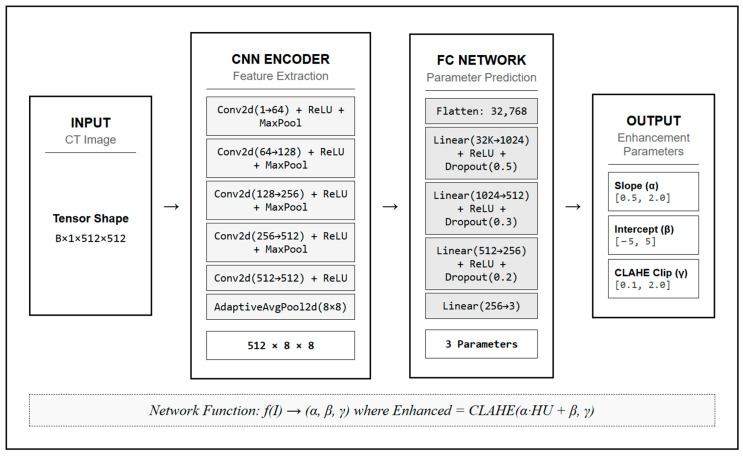
Neural Network Architecture for Dynamic Parameter Optimization in Stroke CT Imaging.

To enhance tissue contrast, we developed a neural network to dynamically optimize the parameters of a linear HU transformation for each individual scan. The neural network architecture, comprehensively detailed in Figure 2, consisted of a CNN encoder for feature extraction followed by fully connected layers for parameter prediction. The CNN encoder included five convolutional layers with batch normalization, ReLU activation, and max pooling, followed by adaptive average pooling to 8 × 8 spatial dimensions. The fully connected network comprised four linear layers with ReLU activation and dropout regularization, ultimately outputting three parameters: slope (α), intercept (β), and the CLAHE clip limit (γ) [20].

The transformation was applied using the equation:

(1) $$\overset{`}{HU}=CLAHE\left(\alpha \cdot HU+\beta ,\;\gamma \right)$$

where $\overset{`}{HU}$ represents the transformed Hounsfield Unit values. The parameters α and β, optimized by the neural network, were constrained to the ranges [0.5, 2.0] and [−5, 5], respectively. The CLAHE clip limit γ was constrained to the range [0.1, 2.0] [21].

Following the optimized linear HU transformation, we applied CLAHE to further enhance image visibility and local contrast. CLAHE is particularly effective in medical imaging because it amplifies subtle intensity differences while preventing over-saturation in homogeneous regions. Unlike conventional histogram equalization methods that apply fixed global adjustments, CLAHE adapts locally by dividing the image into small regions (tiles) and redistributing intensity values within each.

The optimization objective was based on a custom loss function that combined Enhancement Measure Estimation (EME), Peak Signal-to-Noise Ratio (PSNR), and parameter regularization.

The combined loss function was defined as:

(2) $$Loss=w_{1}\cdot {EME}_{loss}+w_{2}\cdot {PSNR}_{loss}+\lambda \cdot {Reg}_{loss}$$

where $w_{1}$ and $w_{2}$ are weighting factors chosen through experimental validation to be approximately 0.8 and 0.2, respectively, and λ is a regularization coefficient set to 0.01 to prevent extreme parameter values. The ${EME}_{loss}$ term promotes image enhancement, the ${PSNR}_{loss}$ term preserves image quality, and the ${Reg}_{loss}$ term penalizes extreme parameter values to maintain reasonable ranges. EME was calculated using block-based contrast measurements, while PSNR was computed between original and enhanced images.

This enhancement process was performed on a scan-by-scan basis, allowing the method to adapt to variations introduced by differences in scanner models, acquisition settings, or individual patient characteristics. This adaptability is a crucial step toward reducing the confounding effects of technical and biological variability, which often limit the generalizability of HU-based methods.

Because the contrast between GM and WM evolves during the natural progression of ischemic stroke, we implemented stage-specific models to account for these temporal dynamics. The neural network was trained using datasets stratified by onset-to-scan time windows across all four clinically defined stages. This design enabled the system to learn parameter configurations specifically attuned to the unique imaging characteristics and pathophysiological changes of each stage.

For instance, in the hyperacute phase, where subtle cytotoxic edema can blur GM-WM boundaries [22], the network learned to emphasize early density shifts. Conversely, in the chronic phase, it adapted to the more pronounced structural and density alterations associated with tissue loss and gliosis. By avoiding a one-size-fits-all transformation, this stage-specific framework increased the method’s sensitivity to biologically meaningful changes over time, thereby improving the accuracy of ischemic stage classification. The approach also provides a more clinically interpretable mapping of tissue evolution, aligning the imaging analysis more closely with the establish pathophysiology of stroke.

### 2.5. Training and Validation

To verify our general approach of applying CLAHE after linear transformation, we conducted paired *t*-tests. These tests demonstrated that a prior linear transformation could enhance the performance of subsequent non-linear enhancement methods. We tested this with various non-linear algorithms including Gamma, Power Law, Log, CLAHE, HE, and AHE, and reported statistically significant improvements (*p* < 0.05) in both EME and PSNR metrics.

The models were implemented in Python 3.9.19 using PyTorch 2.2.2 deep learning framework. Training was conducted on a high-performance GPU workstation to accelerate computation and handle the large dataset efficiently. The optimization process involved minimizing the combined loss function (Equation (2)), with regularization techniques applied to reduce overfitting and ensure robustness across different patient cases.

Following neural network training and parameter optimization, we applied the enhanced images to a classification pipeline. Feature extraction was performed using DenseNet121 architecture, which has proven effective in our previous study [21], followed by classification using three machine learning models: Logistic Regression (LR), Support Vector Machine (SVM), and Random Forest (RF). Performance was assessed using accuracy (ACC), positive predictive value (PPV), recall, and F1-score, all reported with standard deviation (±SD).

To demonstrate the value of our neural network-optimized approach, we performed several comparative analyses: (1) Performance comparison of static versus neural network-optimized linear transformation parameters for stroke classification; (2) Performance comparison of static versus neural network-optimized CLAHE enhancement parameters for stroke classification on linearly transformed images; and (3) Parameter ranges for neural network-optimized enhancement methods across different stroke phases with 95% confidence intervals.

Additionally, we evaluated the impact of our enhancement approach on downstream analysis by applying our previously developed detection and segmentation model to images processed through different enhancement pipelines [21]. Finally, we employed Gradient-weighted Class Activation Mapping (GradCAM) to provide visual explanations of model decisions. This technique highlights the regions in the input images that most influenced the classification outcomes, thereby enhancing clinical interpretability and trust in the automated enhancement process.

## 3. Results

This section presents an extensive evaluation of our neural network–optimized enhancement approach for ischemic stroke classification. We begin by reporting baseline performance using non-enhanced images, followed by a systematic assessment of the enhancement process’s impact across multiple temporal stages. The results demonstrate the model’s effectiveness through quantitative metrics, explainability analyses, and results mapping. We also provide visual comparisons and interpretability to support the clinical validation of the enhancement framework.

### 3.1. Baseline Model Performance

The analysis revealed systematic variations in model performance across stroke phases, with logistic regression (LR) and support vector machines (SVMs) consistently outperforming random forests (RFs) across all time periods (Table 1).

In the hyperacute phase, both LR and SVM achieved high accuracy (0.9498 and 0.9518, respectively), with SVM showing a slight edge in precision and F1 score. RF performed less reliably, with an accuracy of 0.8772, reflecting its difficulty in capturing the very subtle changes seen in early ischemia. In the acute phase, LR proved the most effective classifier, reaching an accuracy of 0.9643 accuracy, which is higher than both SVM (0.9431) and RF (0.9146). The balance of recall and F1 score further highlighted LR’s reliability in detecting acute ischemic tissue, while SVM remained competitive but less consistent. In the subacute phase, performance improved across all models, as stroke-related changes became more distinct. LR achieved the highest accuracy (0.9735), closely followed by SVM (0.9712). Both models showed excellent stability, while RF again trailed behind with an accuracy of 0.9211. In the chronic phase, SVM performed best with an accuracy of 0.9698, slightly surpassing LR (0.9579). RF improved slightly in recall but still lagged behind overall (0.9319). These results suggest that SVM is particularly well suited for classifying chronic ischemic changes, where tissue alterations are more clearly defined.

### 3.2. Image Enhancement Effects

Applying a linear transformation (LT) before non-linear enhancement produced clear improvements in both contrast (EME) and image quality (PSNR) across all methods, as shown in Table 2. For example, gamma correction showed an increase in EME from 45.96 to 47.58 (t = 18.95, *p* < 0.001) and in PSNR from 29.74 to 30.12 (t = 21.57, *p* < 0.001). A similar trend was observed with the power law method, where EME rose from 46.33 to 47.95 and PSNR from 28.72 to 29.09, with both improvements being highly significant (*p* < 0.001).

Local contrast enhancement techniques benefited particularly strongly from prior linear transformation. With CLAHE, PSNR improved from 35.16 to 36.10 (t = 29.31, *p* < 0.001), while EME increased from 27.03 to 28.02 (t = 23.36, *p* < 0.001). AHE showed comparable results, with PSNR increasing from 36.43 to 37.96 (t = 21.15, *p* < 0.001) alongside an EME gain from 40.35 to 41.29 (t = 2.74, *p* < 0.05). These results indicate that CLAHE and AHE not only improved contrast but also preserved structural quality more effectively than global methods.

Histogram equalization (HE) also showed strong EME improvement (46.84 to 47.98, t = 20.35, *p* < 0.001), but its PSNR gain was small (27.80 to 27.29, t = 4.93, *p* < 0.05), suggesting that while it enhances contrast, it may do so at the expense of increased noise.

### 3.3. Neural Network Optimization

**Figure 3:**
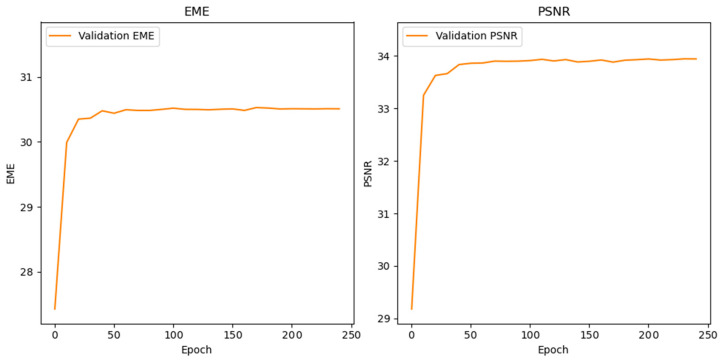
Neural Network Training Convergence of EME and PSNR Metrics During 250-Epoch Enhancement Parameter Learning.

Table 3 compares static and neural network–optimized linear transformation (LT) parameters, showing consistent gains in classification performance across most stroke phases with the optimized approach. The training of the optimization network was stable for both LT parameters. As shown in Figure 3, the validation loss improved steadily over 250 epochs in parallel with increases in EME and PSNR. The curves plateaued at approximately 150 epochs without divergence between training and validation, indicating effective learning of enhancement parameters without overfitting. This stability supports the reliability of the parameters used in subsequent classification experiments.

In the hyperacute phase, logistic regression (LR) performance was essentially unchanged between static and deep LT (accuracy 0.9462 versus 0.9475), whereas support vector machines (SVMs) benefited meaningfully, improving from 0.9478 to 0.9554. Random forests (RFs) showed a small decrease from 0.8840 to 0.8710, suggesting that ensemble methods are less suited to characterizing very early ischemic changes where HU differences are minimal.

During the acute phase, both LR and SVM improved with deep LT. SVM accuracy increased from 0.9548 to 0.9754, and LR rose from 0.9766 to 0.978. RF also improved markedly, from 0.9230 with static LT to 0.9442 with deep LT, with parallel improvements in precision, recall, and F1 score. These findings indicate that optimized parameters enhance both linear and nonlinear classifiers when attenuation differences become more discernible yet still require precise scaling.

In the subacute phase, all models performed strongly under both conditions, reflecting clearer gray–white matter separation. SVM achieved the highest accuracy overall, increasing slightly from 0.9786 to 0.9791. LR improved from 0.9730 to 0.9762. RF remained essentially stable (0.9292 versus 0.9293), suggesting limited additional benefit when tissue contrast is already well defined.

In the chronic phase, the advantage of deep LT was again apparent. LR increased from 0.9683 to 0.9703, and SVM reached 0.9804 compared with 0.9797 under static LT. RF accuracy was unchanged at 0.9428, although precision and recall were stable. The neural network–optimized LT delivered consistent, albeit sometimes modest, gains. These improvements were most notable in the hyperacute and acute stages, where intensity differences pose the greatest challenge. LR and SVM derived the largest benefits, with SVM emerging as the strongest performer across stages. RF remained less responsive to optimization. These results support adaptive parameter optimization as a means to improve the robustness and responsiveness of classifiers to the dynamic imaging characteristics of ischemic stroke.

As shown in Table 4, a comparison of static CLAHE with neural network–optimized CLAHE (referred to as deep CLAHE) demonstrates that adaptive parameter optimization improves stroke-stage classification across all phases, with the largest gains occurring in the hyperacute, acute, and subacute periods.

In the hyperacute phase, accuracy increased for all classifiers after optimization: logistic regression (LR) rose from 0.9754 to 0.9838, support vector machine (SVM) from 0.9777 to 0.9844, and random forests (RFs) from 0.9403 to 0.9481. This indicates enhanced sensitivity to very early, low-contrast parenchymal changes. During the acute phase, deep CLAHE further improved performance (LR 0.9848 to 0.9889; SVM 0.9873 to 0.9904; RF 0.9598 to 0.9618), supporting the need for precise local contrast amplification as hypodensity becomes more discernible.

Subacute cases showed the most noticeable effect of optimization: LR increased from 0.9825 to 0.9948, SVM from 0.9812 to 0.9919, and RF from 0.9295 to 0.9676. This reflects the advantage of tuning clip limits to emphasize well-formed lesions while avoiding over-enhancement. In the chronic phase, performance approached ceiling levels with deep CLAHE (LR 0.9810 to 0.9901; SVM 0.9808 to 0.9979; RF 0.9587 to 0.9677), consistent with stable attenuation differences in established infarcts.

Deep CLAHE outperformed static CLAHE for every classifier and stage, with the greatest absolute gains from the acute to subacute transition. SVM emerged as the strongest model, achieving near-perfect accuracy in chronic stroke (0.9979), while LR showed consistent improvements across all phases. Although RF remained comparatively weaker, it benefited noticeably from optimization, particularly in subacute and chronic stages. These results indicate that neural network–optimized CLAHE provides a robust, adaptive enhancement strategy that improves both accuracy and stability by balancing local contrast gains with effective noise control.

**Figure 4:**
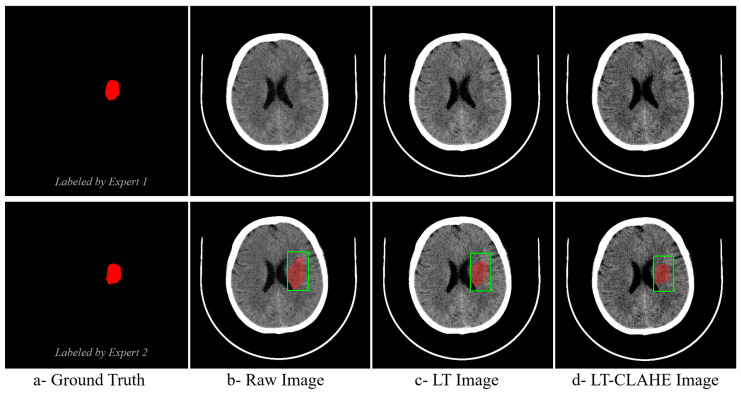
Visual Comparison of Enhancement Methods for Hyperacute Stroke Detection with Expert Ground Truth Annotations. The green square denotes the clinically suspected region of interest (ROI). The red overlay is the delineated/segmented lesion.

The effectiveness of neural network-optimized enhancement is clearly demonstrated through visual comparison. Figure 4 presents a representative hyperacute stroke case showing expert ground truth annotations from two independent radiologists alongside the same case processed as (b) raw image, (c) deep linear transformation (LT), and (d) combined deep LT-CLAHE enhancement. The progressive improvement in lesion visibility and contrast is evident across the enhancement methods, with the LT-CLAHE approach providing the clearest delineation of the subtle hypodense region identified by both experts. This visual enhancement directly correlates with the improved classification accuracies observed in the quantitative analysis, demonstrating how neural network optimization enhances detection of challenging hyperacute stroke lesions.

### 3.4. Parameter Analysis

As summarized in Table 5, the model selected moderate enhancement parameters for hyperacute images, with a slope ranging from 1.249 to 1.253 and a clip limit between 1.054 and 1.061. These conservative values reflect a careful approach aimed at gently improving the visibility of subtle lesions while minimizing the potential for false positives.

For acute cases, the model’s enhancement parameters remained similar, with a slope of 1.250 to 1.257 and a clip limit of 1.050 to 1.058. This consistency indicates that a moderate level of enhancement continues to be appropriate for distinguishing the emerging lesion from normal tissue while guarding against noise amplification.

In the subacute stage, the model identified that higher enhancement is beneficial, as reflected in the highest slope (1.290 to 1.292) and clip limit (1.088 to 1.090) values among all stages. This intensification ensures the now-prominent lesion stands out more clearly, facilitating easier identification at a stage where the risk of amplifying noise is reduced.

For chronic cases, the model appropriately reduced the enhancement parameters, with a slope of 1.240 to 1.258 and a clip limit of 0.941 to 0.959. This restrained approach preserves the integrity of the already clear lesion boundaries, avoiding the potential to exaggerate chronic changes or normal anatomical variations.

### 3.5. Model Interpretability

**Figure 5:**
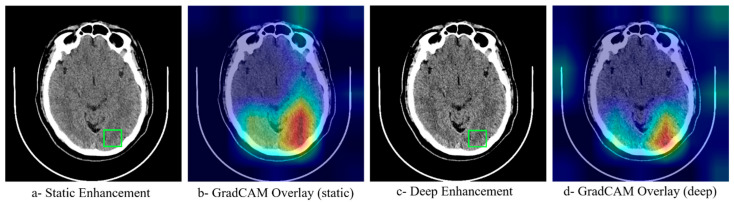
GradCAM Interpretability Analysis Comparing Attention Maps Between Static and Neural Network-Optimized Enhancement Methods. The green square denotes the clinically suspected ROI. Heatmap colors represent the level of model attention, where red/yellow indicate high activation and blue/green indicate low activation regions.

To understand how neural network optimization influences model decision-making, GradCAM activation maps were generated for both static and deep enhancement approaches. Figure 5 reveals that models trained on neural network-optimized images demonstrate more focused attention on clinically relevant regions, with activation patterns closely matching the anatomical locations of ischemic tissue. The optimized enhancement methods produced more coherent and interpretable activation maps compared to static parameters, suggesting that the improved classification performance stems from better feature extraction rather than artifact amplification. This interpretability analysis supports the clinical applicability of the deep enhancement approach.

**Figure 6:**
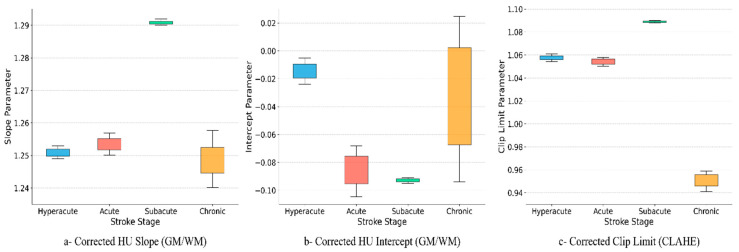
Stage-specific optimization of HU and CLAHE parameters across stroke phases.

Figure 6 illustrates the stage-specific optimization of HU and CLAHE parameters across stroke phases. In the left panel (HU slope), the model maintains moderate contrast scaling in the hyperacute and acute stages (approximately 1.25), increases it in the subacute stage (approximately 1.29), when lesions are most visible, and then reduces it in the chronic stage (approximately 1.24–1.26) since boundaries are already clear and require less enhancement.

In the middle panel (HU intercept), values sit slightly below zero in hyperacute and decrease further in the acute and subacute stages (approximately −0.08 to −0.09), corresponding to increased hypodensity. In the chronic stage, the intercept shifts toward zero with greater variability, reflecting case-specific differences resulting from gliosis and tissue loss.

In the right panel (CLAHE clip limit), local contrast is kept conservative early (≈1.05–1.06), increased in the subacute stage (≈1.09) to accentuate the well-formed lesion, and reduced in the chronic stage (≈0.94–0.96) to avoid over-sharpening long-standing changes.

These parameter trends align with stroke biology: minimal enhancement early to avoid amplifying noise, intensification in the subacute phase to highlight the lesion, and reduction in the chronic phase to preserve clean margins. The tighter confidence intervals in hyperacute and subacute stages suggest consistent model choices across patients, while chronic cases show more natural variability.

## 4. Discussion

Our findings across all analyses confirm that optimized Hounsfield Unit (HU) transformations and adaptive enhancement techniques significantly improve the classification of ischemic stroke in CT imaging. The results show that logistic regression (LR) and support vector machines (SVMs) consistently achieve high accuracy across all stroke phases, while random forests (RFs) perform less reliably. LR demonstrated particular strength in the acute and subacute phases, where attenuation differences between gray and white matter are subtle and evolving, whereas SVM achieved the best performance in the chronic phase, where tissue alterations are more distinct. This highlights the importance of selecting classifiers that are sensitive to the temporal dynamics of stroke progression rather than applying a uniform approach.

These results align with previous HU-based work by Mokin et al. and Reidler et al. [10,13], which showed improved early detection using attenuation-based metrics, albeit in single-center, retrospective studies. Similarly, Said et al. demonstrated that stage-specific HU thresholds can reliably distinguish between phases, but their reliance on fixed thresholds limited reproducibility [14]. Our study builds on these efforts by combining machine learning with optimized HU transformations to capture subtle temporal variations and improve classification robustness.

The addition of a linear transformation prior to non-linear enhancement significantly improved both contrast (EME) and structural fidelity (PSNR) across all methods. Global techniques such as gamma correction and power law demonstrated marked gains in EME and PSNR, consistent with Said et al. [14], who reported improved staging accuracy with HU thresholding. However, histogram equalization, while increasing EME, showed minimal benefit in PSNR, reflecting a known trade-off of contrast enhancement at the expense of noise amplification, as described by Lev et al. In contrast, adaptive methods such as CLAHE and AHE achieved more balanced improvements in both metrics [15]. This finding supports Yao et al., who demonstrated that preserving local textural features enhances radiomics-based models for onset-time classification [18]. Similarly, Inamdar et al. emphasized the importance of preserving structural details in preprocessing for deep learning [19]. Our results extend these insights by showing that an optimized linear transformation combined with adaptive non-linear enhancement provides a reproducible and clinically meaningful pipeline for temporal stroke staging.

The comparison of static versus neural network–optimized linear transformations (deep LT) further demonstrated the benefits of dynamic parameterization. Deep LT improved performance most in the hyperacute and acute stages when subtle attenuation differences pose the greatest classification challenge. For example, SVM accuracy in the acute phase increased from 0.9548 with static LT to 0.9754 with deep LT, and RF accuracy improved from 0.9230 to 0.9442. These findings are consistent with Reidler et al., who showed that HU-based changes are highly parameter-dependent, reinforcing the value of adaptive optimization [13]. In later stages, improvements were smaller but still consistent, supporting the use of dynamic LT for reproducibility and generalizability across diverse patient populations and scanners.

Neural network–optimized CLAHE (deep CLAHE) showed even stronger effects, particularly in subacute and chronic stages. Accuracy rose from 0.9825 to 0.9948 for LR and from 0.9812 to 0.9919 for SVM in the subacute phase. In chronic stroke, SVM achieved near-perfect performance (0.9979) compared to 0.9808 under static CLAHE. These improvements reflect the ability of adaptive CLAHE to preserve lesion boundaries without amplifying noise, a key limitation of global enhancement methods. Compared with threshold-based HU staging [6,12,14]. and heuristic-based ASPECTS scoring [4,10], our results show that neural network-driven optimization offers a reproducible, automated solution. While advanced deep learning models such as those of Inamdar et al. can achieve similar accuracies, they often require large-scale training data and lack transparency. In contrast, our approach achieves comparable performance with lightweight, interpretable classifiers, making it more feasible for real-world integration.

The analysis of enhancement parameter ranges across stages (Table 5) revealed that the model adapted settings in accordance with stroke pathophysiology. Conservative slopes and clip limits were selected in hyperacute and acute stages, where aggressive enhancement risks amplifying noise and artifacts. In the subacute stage, parameters were maximized to accentuate stronger lesion signals. In the chronic stage, settings were reduced again to avoid exaggerating established changes. This stage-specific adaptation is consistent with earlier observations from Lev et al. and Said et al. [14,15], who highlighted the limitations of static thresholds and fixed windowing. By dynamically calibrating enhancement parameters, our approach minimizes false positives in early strokes, maximizes conspicuity in subacute cases, and preserves lesion integrity in chronic presentations.

The visual comparison in Figure 4 demonstrates the practical effect of neural network–optimized enhancement on hyperacute stroke detection. Relative to the raw image and the image processed with deep linear transformation alone, the combined deep LT–CLAHE output provides the clearest delineation of the subtle hypodense region identified independently by two radiologists. This qualitative improvement is concordant with the quantitative gains observed in classification accuracy, particularly in the hyperacute phase where lesion conspicuity is lowest. The finding is consistent with prior reports that careful intensity manipulation improves early ischemic detection on non-contrast CT, provided contrast is increased without amplifying noise [13]. Our data extend these observations by showing that learned, image-adaptive parameters deliver this balance more reliably than fixed settings.

Model interpretability analyses further support a mechanistic link between enhancement and improved decision making. Gradient- weighted Class Activation Mapping (Grad-CAM) activation maps (Figure 5) are more spatially focused and anatomically concordant with ischemic territories when the models are trained on neural network–optimized images, compared with static enhancement. The optimized pipeline concentrates attention within ASPECTS regions corresponding to the radiological ground truth, suggesting that performance gains are driven by better extraction of clinically relevant features rather than by artifact amplification. This behavior aligns with imaging studies where optimized preprocessing or texture preservation improves both classifier focus and downstream accuracy [12,18], and it supports the broader utility of Grad-CAM for verifying that models attend to disease-specific structures in medical images [23].

As shown in Figure 6, the learned parameters vary systematically by temporal stage, mirroring stroke pathophysiology. The HU slope remains moderate in hyperacute and acute cases, increases in subacute scans where edema and parenchymal hypodensity are well formed, and decreases again in chronic imaging where margins are naturally clear. Intercepts are slightly negative in hyperacute and become more negative in acute and subacute phases, consistent with increasing hypodensity, then drift toward zero in chronic cases where gliosis and volume loss introduce greater inter-case variability. CLAHE clip limits are conservative early, peak in the subacute stage to accentuate lesion–background separation and are reduced in chronic scans to avoid over-sharpening established changes. These findings align with reports that attenuation-based metrics are stage dependent and benefit from context-aware parameterization rather than one-size-fits-all thresholds [23,24].

The qualitative gains in lesion conspicuity, the anatomically coherent attention maps, and the stage-aware parameter profiles provide a coherent explanation for the performance improvements observed in our experiments. Learned enhancement increases signal in clinically relevant regions while limiting noise amplification, a balance that classical windowing and fixed histogram methods achieve inconsistently [15]. Although end-to-end deep architecture can also reach high accuracy given large training sets, the present approach delivers comparable benefits with interpretable intermediate steps that can be reviewed and audited, facilitating workflow integration. By coupling optimized HU transformation with adaptive local contrast and by confirming model focus with Grad-CAM, our framework advances beyond earlier threshold-based pipelines and radiomics preprocessing that rely on fixed parameters, offering a reproducible and clinically meaningful strategy for stage-specific stroke assessment.

Our study provides strong evidence that neural network–optimized HU transformations and adaptive enhancement methods address major gaps in the stroke imaging literature. Prior studies have largely been retrospective, single-center, and reliant on static thresholds or heuristic settings, limiting reproducibility. By integrating adaptive preprocessing into interpretable models such as LR and SVM, our study demonstrates that it is possible to achieve robust, reproducible, and clinically meaningful classification across all temporal stages of ischemic stroke. This stage-aware approach represents a practical bridge between experimental imaging research and real-world clinical workflows, particularly in emergency settings where rapid, reliable decision-making is critical.

## 5. Conclusions

Neural network–optimized Hounsfield Unit transformations combined with adaptive enhancement, particularly optimized CLAHE, improved CT-based classification of ischemic stroke across temporal stages. Logistic regression and support vector machines consistently outperformed random forests; LR was strongest in the acute and subacute phases, whereas SVM approached ceiling performance in chronic stroke. Gains were most noticeable from hyperacute to subacute imaging, where subtle attenuation differences benefit from data-driven tuning.

The approach is interpretable and physiologically plausible. Grad-CAM demonstrated model focus within clinically relevant regions, and the learned parameters (HU slope, intercept, CLAHE clip limit) varied across stages in line with expected changes in tissue contrast. Training converged stably with parallel improvements in image-quality metrics, supporting the reliability of the optimized settings. Relative to fixed thresholds or heuristic windowing, stage-aware optimization improved lesion conspicuity when changes were faint and preserved margins when lesions were well defined, making the pipeline suitable for emergency workflows where rapid, confident decisions are essential.

Future work should extend validation across multiple centers, include prospective-reader studies, and correlate imaging predictions with functional outcomes to confirm generalizability and clinical impact.

## 6. Limitations and Future Work

Several limitations should be acknowledged. Our data were collected from a single institution with cross-validation performed at the slice level rather than patient level, which may yield optimistic performance estimates. While we intentionally included three different scanners to introduce technical heterogeneity, the cohort of 68 patients is relatively modest, and the retrospective design limits assessment of clinical workflow impact. Comorbidity data and standardized clinical severity scores such as NIHSS were not systematically available, limiting the ability to correlate imaging parameters with functional outcomes. Normal controls were not formally age- or sex-matched, which may introduce demographic bias.

Future work should prioritize prospective multi-center validation with patient-level stratification to confirm generalizability across diverse healthcare settings. External validation on independent datasets, integration with state-of-the-art architectures, and reader studies correlating imaging predictions with functional outcomes would establish clinical utility. Real-time workflow integration studies would demonstrate practical applicability for emergency stroke assessment and support translation from research settings to routine clinical practice.

## Figures and Tables

**Table 1:** Performance Analysis of Classification Models on Original Non-Enhanced Stroke Dataset Across Different Stroke Phases.

Dataset	Classifier	ACC (±SD)	Precision (±SD)	Recall (±SD)	F1 (±SD)
Hyperacute	LR	0.9498 ± 0.0057	0.9498 ± 0.0058	0.9498 ± 0.0057	0.9496 ± 0.0057
	SVM	0.9518 ± 0.0068	0.9537 ± 0.0061	0.9518 ± 0.0068	0.9510 ± 0.0071
	RF	0.8772 ± 0.0069	0.8937 ± 0.0096	0.8792 ± 0.0070	0.8728 ± 0.0069
Acute	LR	0.9643 ± 0.0148	0.9646 ± 0.0146	0.9643 ± 0.0148	0.9643 ± 0.0149
	SVM	0.9431 ± 0.0146	0.9467 ± 0.0130	0.9431 ± 0.0146	0.9428 ± 0.0147
	RF	0.9146 ± 0.0092	0.9288 ± 0.0086	0.9235 ± 0.0099	0.9211 ± 0.0083
Subacute	LR	0.9735 ± 0.0047	0.9736 ± 0.0047	0.9735 ± 0.0047	0.9735 ± 0.0047
	SVM	0.9712 ± 0.0072	0.9721 ± 0.0071	0.9712 ± 0.0072	0.9709 ± 0.0073
	RF	0.9211 ± 0.0092	0.9278 ± 0.0138	0.9218 ± 0.0139	0.9222 ± 0.0110
Chronic	LR	0.9579 ± 0.0114	0.9581 ± 0.0113	0.9579 ± 0.0114	0.9579 ± 0.0114
	SVM	0.9698 ± 0.0085	0.9701 ± 0.0084	0.9698 ± 0.0085	0.9698 ± 0.0085
	RF	0.9319 ± 0.0092	0.9263 ± 0.0109	0.9392 ± 0.0048	0.9323 ± 0.0066

**Table 2:** Paired *t*-test Results Comparing Original and Linearly Transformed Images for Each Non-Linear Enhancement Method.

Method	EME				PSNR			
	Mean_t0_	Mean_t1_	*t*-Statistic	*p*-Value	Mean_t0_	Mean_t1_	*t*-Statistic	*p*-Value
Gamma	45.96	47.58	18.95	<0.001	29.74	30.12	21.57	<0.001
Power Law	46.33	47.95	18.72	<0.001	28.72	29.09	20.33	<0.001
Log	42.29	43.95	17.70	<0.001	16.51	17.06	28.59	<0.001
CLAHE	27.03	28.02	23.36	<0.001	35.16	36.10	29.31	<0.001
HE	46.84	47.98	20.35	<0.001	27.80	27.29	4.93	<0.05
AHE	40.35	41.29	2.74	<0.05	36.43	37.96	21.15	<0.001

**Note**: Mean_t0_ = mean values for original images, Mean_t1_ = mean values after LT enhancement.

**Table 3:** Performance Comparison of Static versus Neural Network–Optimized Linear Transformation Parameters for Stroke Classification.

Dataset	Classifier	ACC (±SD)	PPV (±SD)	Recall (±SD)	F1 (±SD)
**Static LT:**					
Hyperacute	LR	0.9462 ± 0.0101	0.9461 ± 0.0102	0.9462 ± 0.0101	0.9460 ± 0.0102
	SVM	0.9478 ± 0.0135	0.9503 ± 0.0126	0.9478 ± 0.0135	0.9468 ± 0.0139
	RF	0.8840 ± 0.0172	0.8921 ± 0.0168	0.8848 ± 0.0167	0.8767 ± 0.0129
Acute	LR	0.9766 ± 0.0101	0.9767 ± 0.0100	0.9766 ± 0.0101	0.9766 ± 0.0101
	SVM	0.9548 ± 0.0124	0.9578 ± 0.0108	0.9548 ± 0.0124	0.9546 ± 0.0126
	RF	0.9230 ± 0.0123	0.9310 ± 0.0124	0.9174 ± 0.0168	0.9258 ± 0.0153
Subacute	LR	0.9730 ± 0.0029	0.9731 ± 0.0028	0.9730 ± 0.0029	0.9730 ± 0.0030
	SVM	0.9786 ± 0.0075	0.9793 ± 0.0070	0.9786 ± 0.0075	0.9784 ± 0.0077
	RF	0.9292 ± 0.0233	0.9341 ± 0.0192	0.9366 ± 0.0213	0.9299 ± 0.0204
Chronic	LR	0.9683 ± 0.0041	0.9683 ± 0.0041	0.9683 ± 0.0041	0.9683 ± 0.0041
	SVM	0.9797 ± 0.0070	0.9800 ± 0.0068	0.9797 ± 0.0070	0.9797 ± 0.0070
	RF	0.9428 ± 0.0171	0.9452 ± 0.0190	0.9413 ± 0.0163	0.9432 ± 0.0192
**Deep LT:**					
Hyperacute	LR	0.9475 ± 0.0096	0.9475 ± 0.0098	0.9475 ± 0.0097	0.9475 ± 0.0097
	SVM	0.9554 ± 0.0124	0.9575 ± 0.0125	0.9554 ± 0.0122	0.9549 ± 0.0128
	RF	0.8710 ± 0.0152	0.8918 ± 0.0158	0.8710 ± 0.0160	0.8643 ± 0.0156
Acute	LR	0.9775 ± 0.0097	0.9783 ± 0.0091	0.9779 ± 0.0089	0.9780 ± 0.0093
	SVM	0.9754 ± 0.0120	0.9766 ± 0.0116	0.9754 ± 0.0112	0.9754 ± 0.0114
	RF	0.9442 ± 0.0120	0.9480 ± 0.0118	0.9446 ± 0.0123	0.9476 ± 0.0122
Subacute	LR	0.9762 ± 0.0017	0.9760 ± 0.0018	0.9761 ± 0.0018	0.9760 ± 0.0018
	SVM	0.9791 ± 0.0044	0.9793 ± 0.0045	0.9790 ± 0.0044	0.9792 ± 0.0046
	RF	0.9293 ± 0.0225	0.9348 ± 0.0205	0.9360 ± 0.0204	0.9302 ± 0.0204
Chronic	LR	0.9703 ± 0.0032	0.9702 ± 0.0031	0.9702 ± 0.0031	0.9702 ± 0.0031
	SVM	0.9804 ± 0.0062	0.9802 ± 0.0061	0.9802 ± 0.0061	0.9802 ± 0.0061
	RF	0.9428 ± 0.0170	0.9450 ± 0.0188	0.9418 ± 0.0160	0.9434 ± 0.0193

**Note**: Static LT uses fixed slope and intercept parameters (a, b) from previous studies, while Deep LT uses neural network-optimized parameters.

**Table 4:** Performance Comparison of Static versus Neural Network–Optimized CLAHE Enhancement Parameters for Stroke Classification.

Dataset	Classifier	ACC (±SD)	PPV (±SD)	Recall (±SD)	F1 (±SD)
**Static CLAHE:**					
Hyperacute	LR	0.9754 ± 0.0092	0.9754 ± 0.0092	0.9755 ± 0.0092	0.9755 ± 0.0092
	SVM	0.9777 ± 0.0061	0.9782 ± 0.0058	0.9777 ± 0.0061	0.9777 ± 0.0061
	RF	0.9403 ± 0.0084	0.9392 ± 0.0079	0.9347 ± 0.0074	0.9350 ± 0.0104
Acute	LR	0.9848 ± 0.0020	0.9849 ± 0.0020	0.9848 ± 0.0020	0.9848 ± 0.0020
	SVM	0.9873 ± 0.0027	0.9875 ± 0.0026	0.9873 ± 0.0027	0.9872 ± 0.0027
	RF	0.9598 ± 0.0060	0.9598 ± 0.0055	0.9598 ± 0.0047	0.9598 ± 0.0041
Subacute	LR	0.9825 ± 0.0026	0.9824 ± 0.0029	0.9824 ± 0.0029	0.9824 ± 0.0028
	SVM	0.9812 ± 0.0046	0.9816 ± 0.0045	0.9815 ± 0.0046	0.9815 ± 0.0046
	RF	0.9295 ± 0.0180	0.9326 ± 0.0142	0.9345 ± 0.0130	0.9312 ± 0.0132
Chronic	LR	0.9810 ± 0.0042	0.9811 ± 0.0040	0.9812 ± 0.0040	0.9811 ± 0.0040
	SVM	0.9808 ± 0.0019	0.9806 ± 0.0022	0.9804 ± 0.0019	0.9805 ± 0.0021
	RF	0.9587 ± 0.0120	0.9583 ± 0.0126	0.9586 ± 0.0128	0.9584 ± 0.0125
**Deep CLAHE:**					
Hyperacute	LR	0.9838 ± 0.0054	0.9840 ± 0.0054	0.9838 ± 0.0054	0.9838 ± 0.0054
	SVM	0.9844 ± 0.0080	0.9847 ± 0.0078	0.9844 ± 0.0080	0.9844 ± 0.0080
	RF	0.9481 ± 0.0141	0.9466 ± 0.0118	0.9487 ± 0.0151	0.9462 ± 0.0193
Acute	LR	0.9889 ± 0.0018	0.9888 ± 0.0018	0.9889 ± 0.0018	0.9888 ± 0.0018
	SVM	0.9904 ± 0.0026	0.9905 ± 0.0024	0.9904 ± 0.0022	0.9904 ± 0.0022
	RF	0.9618 ± 0.0097	0.9631 ± 0.0112	0.9618 ± 0.0128	0.9613 ± 0.0150
Subacute	LR	0.9948 ± 0.0029	0.9949 ± 0.0029	0.9948 ± 0.0029	0.9948 ± 0.0030
	SVM	0.9919 ± 0.0036	0.9920 ± 0.0035	0.9919 ± 0.0036	0.9919 ± 0.0036
	RF	0.9676 ± 0.0097	0.9688 ± 0.0112	0.9639 ± 0.0128	0.9656 ± 0.0150
Chronic	LR	0.9901 ± 0.0060	0.9902 ± 0.0060	0.9901 ± 0.0060	0.9901 ± 0.0060
	SVM	0.9979 ± 0.0019	0.9979 ± 0.0019	0.9979 ± 0.0019	0.9979 ± 0.0019
	RF	0.9677 ± 0.0115	0.9676 ± 0.0116	0.9719 ± 0.0102	0.9698 ± 0.0110

**Note**: Static CLAHE uses fixed clip limit values from previous studies, while Deep CLAHE uses neural network-optimized clip limit values.

**Table 5:** Parameter Ranges for Neural Network-Optimized Enhancement Methods Across Different Stroke Phases.

Dataset	Parameters Ranges 95% CI					
	Slope		Intercept		Clip Limit	
	LB	UP	LB	UP	LB	UP
Hyperacute	1.249	1.253	−0.024	−0.005	1.054	1.061
Acute	1.250	1.257	−0.105	−0.068	1.050	1.058
Subacute	1.290	1.292	−0.095	−0.091	1.088	1.090
Chronic	1.240	1.258	−0.096	0.025	0.941	0.959

**Note**: LB = Lower Bound, UB = Upper Bound, CI = Confidence Interval.

## Data Availability

The data presented in this study are available on request from the corresponding author, subject to appropriate ethical approvals and data sharing agreements.
